# Evolutionary Insights from Association Rule Mining of Co-Occurring Mutations in Influenza Hemagglutinin and Neuraminidase

**DOI:** 10.3390/v16101515

**Published:** 2024-09-25

**Authors:** Valentina Galeone, Carol Lee, Michael T. Monaghan, Denis C. Bauer, Laurence O. W. Wilson

**Affiliations:** 1 Institute of Computer Science, Freie Universität Berlin, 14195 Berlin, Germany; valentina_galeone@outlook.it; 2 Australian e-Health Research Centre, Commonwealth Scientific and Industrial Research Organisation, Sydney, NSW 2145, Australia; carol.lee@csiro.au (C.L.); denis.bauer@csiro.au (D.C.B.); 3 Institute of Biology, Freie Universität Berlin, 14195 Berlin, Germany; michael.monaghan@igb-berlin.de; 4 Leibniz Institute of Freshwater Ecology and Inland Fisheries (IGB), 12587 Berlin, Germany; 5 Department of Biomedical Sciences, Macquarie University, Sydney, NSW 2109, Australia

**Keywords:** influenza, H3N2, association rule mining, antigenic drift, co-occurring mutations

## Abstract

Seasonal influenza viruses continuously evolve via antigenic drift. This leads to recurring epidemics, globally significant mortality rates, and the need for annually updated vaccines. Co-occurring mutations in hemagglutinin (HA) and neuraminidase (NA) are suggested to have synergistic interactions where mutations can increase the chances of immune escape and viral fitness. Association rule mining was used to identify temporal relationships of co-occurring HA–NA mutations of influenza virus A/H3N2 and its role in antigenic evolution. A total of 64 clusters were found. These included well-known mutations responsible for antigenic drift, as well as previously undiscovered groups. A majority (41/64) were associated with known antigenic sites, and 38/64 involved mutations across both HA and NA. The emergence and disappearance of N-glycosylation sites in the pattern of N-X-[S/T] were also identified, which are crucial post-translational processes to maintain protein stability and functional balance (e.g., emergence of NA:339ASP and disappearance of HA:187ASP). Our study offers an alternative approach to the existing mutual-information and phylogenetic methods used to identify co-occurring mutations, enabling faster processing of large amounts of data. Our approach can facilitate the prediction of critical mutations given their occurrence in a previous season, facilitating vaccine development for the next flu season and leading to better preparation for future pandemics.

## 1. Introduction

Seasonal influenza viruses (Orthomyxoviridae) are responsible for recurring epidemics worldwide, leading to approximately 250,000 to 500,000 deaths each year [1]. Most of these cases are caused by Influenza types A and B. The former circulates in animal hosts (bird and swine) and has caused devastating pandemics, for example, the Spanish Flu (1918) and swine flu (2009) caused by H1N1, and Hong Kong flu (1968) caused by H3N2 [2]. Influenza B primarily infects humans and has been circulated in human populations since the 1940s, having since diverged into two main lineages: B/Victoria and B/Yamagata [3]. Given their significant impact on human health, these two types are the primary targets for existing influenza vaccines. However, despite extensive research, the success of influenza can be attributed to its ongoing evolution and efficient transmission between hosts, allowing it to evade host immunity that results from previous infections or vaccinations [4].

The surface proteins hemagglutinin (HA) and neuraminidase (NA) play critical roles in viral replication and successful infection [5]. The receptor binding site (RBS) in the globular head domain of HA binds onto sialic acids (SA) on the surface of host cells, while NA is responsible for cleaving the HA–SA bond of budding virion for release and infection of new cells [6]. The gradual accumulation of mutations (antigenic drift) in the RBS, namely the five known antigenic regions, can influence host specificity and cell types [7,8,9], constantly challenging the effectiveness of new vaccines. However, the close proximity of NA and HA indicates that immune pressure caused by immunisation can generate favourable mutations in HA or NA to increase specificity and antigenicity or allow efficient release of virions, respectively [5,10,11]. This suggests that the co-evolution of HA and NA mutations leads to enhanced virus transmission and overall viral fitness and outbreaks. As seasonal influenza evolution involves simultaneous mutations, not just gradual single-point changes [12], a method to rapidly detect and analyse mutation groups, establish temporal relationships, and potentially uncover cause-and-effect links would be invaluable to identify important co-occurring mutations in influenza and discover potential functional links.

Many methods have been used to monitor mutation sites under positive selection-driving for or maintaining beneficial mutations, including statistical analysis and machine learning as an alternative to phylogenetics. Reconstructing evolutionary events through phylogenetics (maximum likelihood or Bayesian methods) often requires significant computational resources but allows for a more precise understanding of the chronological order of individual mutations. Association rule mining (ARM) offers a suitable alternative to phylogenetics or methods such as mutual information (MI), which only examines pairwise interactions [13,14,15]. This technique operates on transactional data: a “transaction” represents a set of items (mutations) that occur together frequently or are connected non-randomly and association rules that describe that the relationship between items are generated through the frequency of these itemsets [16,17]. ARM has been used to determine the various contribution of mutations to host range, pandemic/seasonal influenza, and the antigenic evolution of influenza virus [16,18,19] and has been applied to other pathogens and diseases as well [20,21,22,23,24,25]. The popularity and versatility of ARM arise from its capacity to uncover groups of key associations within datasets without demanding significant computational power, thanks to advancements in algorithmic efficiency [26,27]. Furthermore, the results are easily interpretable, making ARM an accessible and powerful tool for data analysis. The potential of this method, particularly when applied to co-occurring mutations in influenza, was first explored by Chen et al. (2016) [16].

Building on the work of [16] this study differs in three key approaches: (1) we limited the dataset to sequences from the year 2005 onwards. This decision was influenced by the increased availability of sequenced viruses in databases due to next generation sequencing (NGS) technologies. Chen et al. [16] collected data for the H3N2 subtype following the year 1968 (Hong Kong flu outbreak), which means that some years had a particularly small number of sequences. Therefore, limiting the range of years allows a notably higher number of sequences to be retained for study, enabling us to detect clusters of simultaneous mutations evolving exactly from one flu season to the next. (2) In our work, criteria used to identify sequences that are evolutionarily close were useful in excluding sequences that may not be genetically related. This enabled us to confidently assess which mutations occurred from one flu season to the next. (3) We included both HA and NA to detect co-evolving mutations, as these two proteins are closely interconnected in terms of function and evolution [28,29].

This study describes the application of ARM to detect co-occurring mutation clusters in the HA and NA of influenza virus A H3N2, combined with network analysis and phylogenetic analysis as a validation step by tracking the associations found by ARM. The aim of this study was to develop a method that can effectively identify patterns of co-evolution within the important HA and NA proteins of influenza A and establish links between functional mutations.

## 2. Materials and Methods

**The datasets:** Data were collected from the Bacterial and Viral Bioinformatics Resource Center (BV-BRC) [30]. The data used in this study included the complete sequences for HA and NA of influenza virus A found within human hosts in North America. The scope was limited to North America to avoid potential biases in the representation of influenza virus strains, as most available sequences were sourced from this region. This encompassed 6915 sequences for the H3N2 subtype, spanning from 2006 to 2020. Sequences with >5% ambiguous characters (i.e., nucleotides other than A, C, G, and T) were removed and then organised into flu seasons based on whether they were collected before or after September 1st of a given year.
**Data pre-processing:** Additional filtering was performed to exclude potential reassorted sequences and retain sequences from H3N2 only. Sequences from consecutive bins were aligned using Clustal Omega, and the distance matrix was calculated using Kimura Two-Parameter (K2P) in the EMBOSS distmat command [31]. Preliminary results indicated that sequences from consecutive flu seasons typically ranged from 1–5 substitutions per 100 amino acids. BLAST [32] was used to confirm the subtype and year, and outliers (>5 substitutions) either matched a different subtype, host, or year contrary to metadata and were eliminated (0.1%) assuming misclassification or as an outcome of reassortment events.

The DNA sequences were translated into amino acid sequences, and where necessary, leading gaps were retrieved from NCBI and appended. Sequences with insertions were removed from the dataset (20 sequences; <0.1%), as our methods do not handle insertions. Custom Python scripts were used to process indels and duplications. The names of the selected strains are provided in Table S1. A summary of the sequences retained after each step is shown in Table 1.

**Association rule mining:** ARM was used to extract co-occurring mutations between and within the HA and NA glycoproteins and their transitions between consecutive flu seasons. A modified version of the association rule function from mlxtend version 0.21.0 [33] was used to identify itemsets (specific combinations of mutations between flu seasons). This was to generate rules associated with antigenic sites for HA and NA [7,34]. To compute the complete set of all frequent itemsets, the algorithm FPgrowth was used. Default values were used for the calculation of frequent itemsets (minimum support value = 0.05) and association rules (minimum confidence = 0.5). In practical terms, this meant that all rules were generated with a confidence level higher than 50%. Default values were relatively lenient to allow the generation of sufficient rules and order by the highest confidence.

This process was repeated multiple times to collect N number of transactions. A value of N = 250 performed well, yielding consistent results across different random seeds while maintaining a low probability of randomly selecting the same pair of flu sequences and introducing bias. This formed the basis of downstream analysis to identify patterns of co-occurring mutations over consecutive flu seasons. This final approach involved extracting transactions independently from each pair of flu seasons, characterising these transitions and detecting recurring patterns by comparing the clusters across years. The number of sequences available prior to 2006 at the time of collection consisted of <500 sequences per season. Thus, flu sequences before 2006 were not considered to ensure that the categories included were sufficiently large to reduce the risk of resampling bias when increasing the number of selected sequences. Several metrics were used to measure the strength and confidence of each association (Table S2). We included an additional metric, Zhang’s metric, for a more precise measure of confidence and an extension of Lift [35]. The scale ranged from −1 (complete disassociation) to 1 (complete association). Association rules with a Zhang’s metric > 0.85 were prioritised to focus on robust evidence of association.

To address false or reversed mutations, we assumed that clusters of mutations occur in a single direction (from one flu season to the next, not vice versa) based on the year. Two methods were employed to clarify the order of mutations: (1) phylogenetic analysis, in which we examined the order of mutations to determine their sequential occurrence, and (2) frequency plots, where mutations with the highest frequencies were considered valid, while those appearing in the opposite direction were considered invalid (see Figure S1).

Relevant associations detected between the two flu seasons were visualised through a network displaying co-occurring mutations using Networkx (version 3.1) [36] and Pyvis (version 0.3.1) [37].

**Sequence variability and phylogenetic validation of mutation transitions:** The maximum likelihood (ML) in IQ-TREE [38] was used to construct separate phylogenetic trees for H3N2 hemagglutinin and neuraminidase and establish a mutation threshold for our approach. The FLU amino acid substitution model [39] with the Gamma model was used to account for rate heterogeneity. This model specifically addresses the evolution of influenza virus sequences. The threshold was to exclude pairs belonging to distinct clades of the same influenza type that are not likely to have occurred simultaneously in one year and, thus, discard those exceeding the threshold. In certain years, such as 2012 and 2019, the division in subclades is more evident. A threshold of mutations T = 15 was chosen that was suitable to avoid the alignment of two distantly related sequences that belong to different subclades (Figure S2). Additionally, tree data from IQ-TREE was used to map mutation clusters to validate and cross-reference the identified clusters.
**Shannon’s entropy and frequency plots:** Shannon’s entropy serves as a valuable tool for assessing the variability of amino acid positions within multiple sequence alignment. Multiple sequence alignments for each flu season were used to calculate the entropy values for each position, and the mean value across bins was used to assess the overall variability. Positions with a high average entropy value were indicative of the positions having undergone multiple changes over time, providing valuable insights into protein evolution. Entropy was calculated using the following formula:
  $$H=-\sum_{i=1}^{N}p\left(x_{i}\right)\log_{2}\left(p\left(x_{i}\right)\right).$$
  Frequency plots were used to visualise the amino acid frequencies of positions with the highest entropy over time. Transitions in the same position with the lowest frequency were excluded from the results, and no association rules were generated.

## 3. Results

### 3.1. Association Rules and Clusters of Mutations

A total of 1647 rules of association in H3N2, spanning from 2006 to 2020, with confidence larger than 0.5 and support larger than 0.05, were identified. Further filtering by applying a stringent threshold of Zhang’s metrics > 0.85 was used to identify rules with stronger evidence of association. A total of 64 clusters of mutations were found, representing small sets of mutations ranging from two to seven residues, which evolved together from one flu season to the next, with a mean of about four to five clusters occurring for each transition (Table S3). The majority of clusters found in the H3N2 dataset included amino acids at antigenic sites (41 clusters out of 64), possibly linking amino acids involved in antigenic variation with more distant positions. Furthermore, the clusters comprise mutations found in hemagglutinin and neuraminidase or spanning across both proteins.

**Figure 1:**
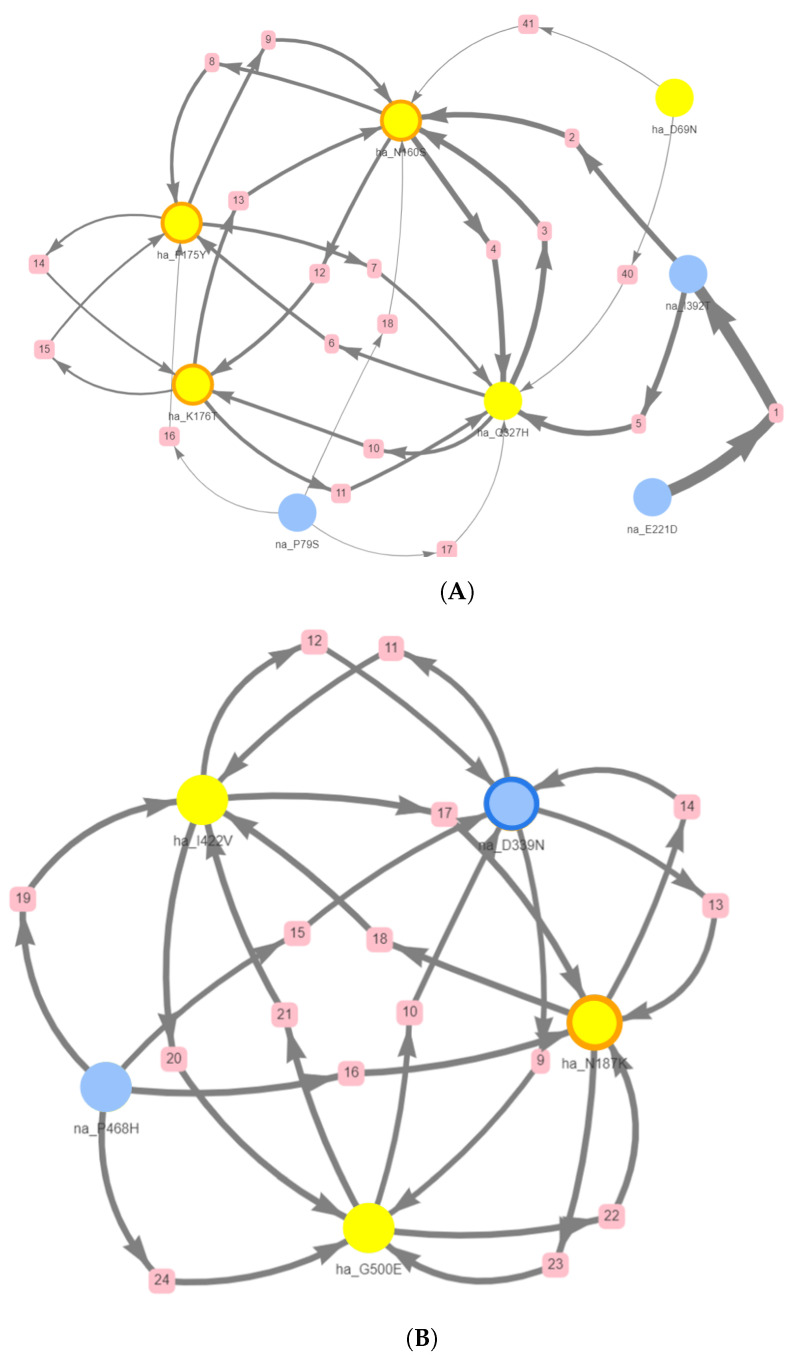
Cluster of mutations in H3N2 during the transition from the (**A**) 2012/13 to 2013/14 and (**B**) 2014/15 to 2015/16 flu season. Transitions include HA and NA antigenic sites. Filled circles indicate mutations in hemagglutinin (yellow) and neuraminidase (blue), and an orange or dark blue border (e.g., node ha_D339N) indicates that the mutation occurred at an antigenic site, pink boxes indicates a (directed) rule identified by a number. The thickness of the edges represents the support of the rule and the direction show the antecedent and consequent.

We visualised the clusters of mutations that included these positions as networks (Figure 1). Each pink box is a (directed) rule identified by a number. These mutation networks not only revealed associations among mutations but also drew attention to noteworthy instances. Notably, we readily identified connections between mutations in the two proteins and specifically within antigenic sites. Figure 1A illustrates the mutation site ha_160 playing a central role in the network and formed connections with HA mutations at positions 69, 175, 176, and 327 and NA mutations at positions 79 and 1392, except for 221, which is individually associated with na_1392. The four HA mutations (160, 175, 176, and 327) are interconnected bidirectionally, suggesting possibilities of A to B and B to A transitions. However, the unidirectional na_I392T to ha_N160S suggests the occurrence of N160S when 392T occurs but not the reverse. Figure 1B shows a similar cluster where antigenic site 339 is interconnected with four other positions.

### 3.2. Shannon’s Entropy and Frequency Plots

**Figure 2:**
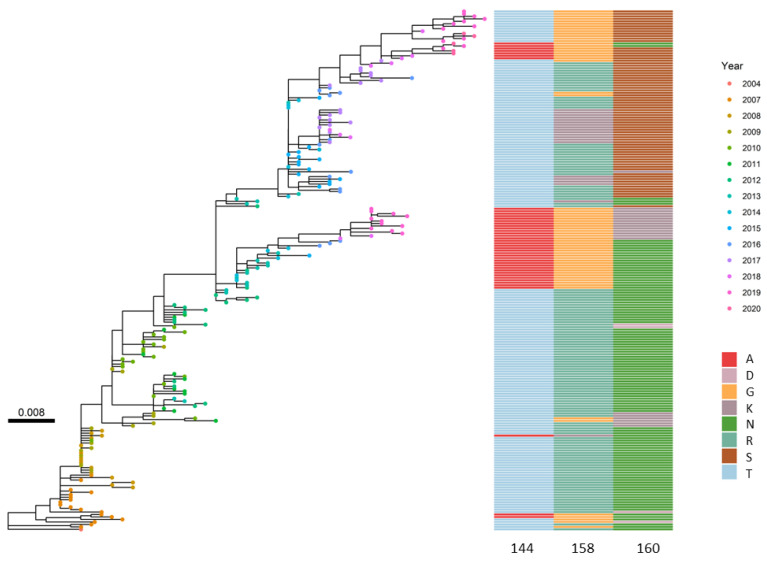
Maximum likelihood phylogenetic tree of 15 randomly selected HA sequences from each flu season with the amino acid illustrated for positions 144, 158, and 160 for each sequence. Coloured dots indicate the year isolated, showing highly temporal relationships between sequences and the protein position with the highest entropy.

Positions with a high average entropy value are indicative of having undergone multiple changes over time, providing valuable insights into protein evolution. For HA, the three positions with the highest entropy were identified as 144, 158, and 160 (Figure S3). These positions are all located within the antigenic regions, and position 158, the one with the highest entropy in the H3N2 dataset, is among the seven key amino acid sites responsible for driving antigenic changes [40]. Phylogenetic analysis (Figure 2) and frequency plots (Figure S4) indicated positions 158 and 144 were found to be strongly associated during both the transition from the flu season 2011/12 to 2012/13 and from 2012/13 to 2013/14 with our method (Figure S3). In both cases, they were not associated with other mutations but formed a cluster consisting of only two elements. However, when examining positions 160 and 144, no clusters demonstrated their association, which was verified with the HA phylogenetic tree (Figure 2). Phylogenetic analysis also showed incongruence between HA and NA, which can be attributed to reassortment events (Figure S5). Notably, the presence of subclades was clear, with hemagglutinin displaying more pronounced subclade formations. In specific cases, such as the years 2012 and 2019, the division into distinct clades was more evident.

### 3.3. N-Glycosylation Sites

An important feature that appears from the results is the emerging and disappearing of N-glycosylation sites, which are crucial post-translational processes that impact the protein’s stability and function by attaching sugar molecules, thereby influencing its biological activity and interactions. For example, in Figure 1B, the two positions in the antigenic sites provided evidence of the emergence of asparagine at position 339 of NA, associated with the disappearance of asparagine at position 187 of HA. The presence of asparagine at these positions suggests the potential formation of N-glycosylation sites. It is noteworthy that among the clusters, 32 out of 64 contain at least one instance of either an emerging or disappearing asparagine. The case of na_D339N and ha_N189K is not unique; in many other instances, an emerging asparagine is coupled with a disappearing one. Another interesting pattern is the emergence of the sequence pattern N-X-[S/T], as in the highlighted cluster occurring during the transition 13/14–14/15, which includes na_S247T and na_S245N. These two mutations have been observed to co-evolve, leading to the creation of an N-glycosylation site at position 245. We can hypothesize that a similar mechanism took place in the clusters listed in Table 2 (full list of clusters in Table S3) that contain mutations na_N465S and na_D463N during the transition 18/19–19/20. In this case, it is reasonable to conclude that mutations 465 and 463 co-evolved, resulting in the formation of a new N-glycosylation site at position 463. Furthermore, the emergence of this new N-glycosylation site is coupled with the potential loss of a site at position 110 of HA.

## 4. Discussion

Research has demonstrated that the antigenic drift of seasonal influenza viruses is not solely driven by gradual single-point mutations but also by simultaneous mutations [12,41]. For this reason, there is a need for methods that are capable of rapidly detecting and analyzing co-occurring groups of mutations, identifying temporal relationships within such groups, reconstructing the order of events underlying major evolutionary changes, and eventually uncovering any cause–effect relationships that may exist among these mutations. These data can be used to establish effective predictive methods for monitoring the emergence of new viral strains that could be more virulent or influence current vaccination protocols. The current study presents a dedicated approach designed to address this initial step by rapidly characterizing groups of simultaneous mutations through the application of association rule mining principles.

Several clusters of co-occurring mutations were found to extend across both hemagglutinin and neuraminidase, suggesting interconnected functionalities between these proteins, a hypothesis that should be better investigated to identify its potential roles in influenza pathogenicity. HA and NA work in tandem to ensure efficient virion release for further infection of host cells [42]. Many of the association rules involved both HA and NA (38/64) and include one to five HA mutations and one to three NA mutations. The functional balance between HA and NA proteins needs to be maintained due to their complementary functions, where the evolutionary potential of HA is influenced by NA in an effort to increase viral fitness through immune escape [5,43]. Mutations in NA can be restricted so as not to impact the epitope binding potential of HA for initial infection, not dissimilar to the non-random reassortment of specific HA and NA subtypes for cross-species infection [5,18]. These co-occurring mutations in both HA and NA further provide insights into NA-independent resistance, where many NA inhibitor-resistant mutants are present due to mutations in HA through reduced binding affinity and reducing the dependency on NA for virion release [44]. This dependency may indicate why NA inhibitor resistance is less prevalent than adamantane (1–4% adults shedding resistant virus versus up to 23%, respectively) [45,46], which targets the M2 protein [47]. The current study did not identify any NA inhibitor-resistant mutations in either HA or NA. However, further studies may benefit from categorising influenza sequences by their antibody affinity to identify rules associated with antiviral resistance.

We also identified clusters linked to the emergence or disappearance of N-glycosylation sites, shedding light on glycosylation-related changes in protein function. The glycosylation of HA and NA is indicative of immune evasion without loss of viral fitness [48]. Our results also highlighted the na_S247T and na_S245N mutation observed circa 2015 with reduced NA antibody binding [49]. This is in concordance with the N-X-[S/T] pattern observed to prevent antibody contact with underlying residues and, thus, impacts vaccine efficacy [50]. The HA and NA proteins function synergistically to successfully infect host cells, and modifications to HA–NA, such as through glycosylation, can impact viral fitness. Our study identified several mutational transitions in the years from 2015–2020, which we found in IAV subtypes that differed from the vaccine strains (A/Singapore/infimh-16-0019/2016). These co-occurring mutations include na_P126L, na_K220N, and na_V303I [51,52,53], in addition to clustering with ha_E78G, ha_K108R, ha_T151K, ha_R158G, and ha_H327Q, which differ from the A/Hong Kong/4801/2014(H3N2) vaccine strain. Interestingly, we did not find na_X329N and na_E344K to be co-occurring, which is often linked to higher neuraminidase-inhibiting (NI) activity. However, this may be due to the decreased percentage of isolates with N-glycosylation at na_329 since 2015 [54]. We also noted the na_L140I and na_V149A mutations differing from the A/Switzerland/8060/2017 vaccine strain used for the southern hemisphere. The latter mutation was also close to the active site and may have influenced sialidase activity [55]. Additionally, we found notable clusters resulting in the loss of glycosylation sites such as ha_N187K from 2014. This co-occurred with other HA mutations (ha_I422V and ha_G500E) commonly found around 2016–2017 [52] and na_P468H and na_339N, with the latter being an emerging glycosylation site. HA and NA have a complex co-evolution dynamic, constantly changing to modulate binding and cleaving activities and have the potential to compensate for function in the other protein [56]. ARM has the potential to extract these complex relationships and identify these frequently interacting sites. The potential of identifying mutations contributing to glycosylation or sequons and evaluating their influence on antibody binding and vaccine efficacy would improve influenza vaccine development through the optimisation of using both HA and NA mutations for consideration.

In the current study, the limited 15-year range excluded insights into mutations that may have occurred multiple times over the earliest sequences available. As a result, only a few mutations recurred in different combinations within the database, with no clear pair or group of mutations exhibiting repeated occurrences. The inclusion of earlier sequences (<2006) with consideration of potential resampling bias could offer insights into more historical trends. Nonetheless, a noticeable pattern that emerged was the consistent appearance and disappearance of asparagine, which potentially represents the emergence and disappearance of N-glycosylation sites. These mutations often occurred in pairs: an N appearing in a new position was coupled with an N disappearing from another position, spanning both the HA and NA proteins.

Association rule mining is a powerful tool to rapidly detect association in a transaction dataset: the efficiency is given by a runtime of less than one minute. Correctly characterising linked mutations and identifying the major determinants that drive their associations is the first critical step in developing an effective tool that can prepare for future pandemics by detecting key groups of associated mutations in time. In addition to ARM, a comprehensive predictive tool for monitoring virus evolution and anticipating future mutations should integrate information from various sources, not solely relying on association rule mining. In silico modelling (e.g., AlphaFold [57]) of mutational transitions identified here can provide further insight into the impact of these mutations on the structure of the proteins and the potential effect on receptor binding and cleavage. Another avenue of exploration is the incorporation of phylogenetic analysis as an integral component rather than using it solely for validation, as was the case here. Such an approach would allow further valuable insights into the evolutionary history of key mutations, particularly those within antigenic sites or receptor-binding sites. By tracking the lineage of these mutations on a phylogenetic tree, deeper insights into their emergence and persistence over time will complement the information obtained through association rule mining regarding more distant mutations associated with these key positions.

## 5. Conclusions

These findings highlight the potential of ARM to identify co-occurring mutations of functional interest. ARM provides a valuable foundation for further analysis and the potential development of predictive tools. ARM can be extended to other influenza subtypes to uncover broader evolutionary patterns and co-occurring mutations that may be implicated in preparedness for future outbreaks and be further developed with predictive algorithms.

## Figures and Tables

**Table 1:** Total number of sequences after each step of the pre-processing workflow.

Step	H3N2
Download data from BV-BRC	13,543
Selecting the data bins	12,865
Removing incomplete sequences	12,622
Removing evolutionary distant sequences	12,612
Removing sequences with insertions	12,604
Removing duplicates	6915

**Table 2:** Subset of co-occurring mutation clusters in H3N2. The asterisks (*) indicate that the mutation occurred at an antigenic site.

Flu Season	HA Mutations	NA Mutations
07/08–08/09	N160*K, N205K, V229*A, K174*N, E78*K	-
12/13–13/14	F175*Y, Q327H, D69N, K176*T, N160*S	E221D, P79S, I392T
13/14–14/15	-	S247T, S245N
15/16–16/17	I422V, G500E, N187*K	N339*N
18/19–19/20	K99E, I538M, Y110N	N465S, D463N, G346*D

## Data Availability

All custom Python scripts for the ARM analyses are available in Github: https://github.com/valegale/influenza_mutations (v1.0.0), and data pre-processing scripts can be found at https://github.com/valegale/preprocessing_influenza (v1.0.0).

## Supplementary Materials

The following supporting information can be downloaded at https://www.mdpi.com/article/10.3390/v16101515/s1, Table S1: Name of strains used in this study; Table S2: Description and formula of metrics used to evaluate the association rules generated in this study; Table S3: Summary of the 64 co-occurring mutation clusters in H3N2. Figure S1: Illustration of potential ‘reverse mutations’, where sequence 1 and 2 are randomly selected from two consecutive flu seasons (bins) with sequence 2 from a more recent bin; Figure S2: Boxplot showing the different number of mutations when the sequences belong to the same clade compared to belonging to different clades in (a) 2012 and (b) 2019; Figure S3: Positions with a high entropy showed a larger number of amino acids in the frequency plots; Figure S4: Frequency plots of amino acid positions depicting residue frequency for each flu season; Figure S5: H3N2 maximum likelihood phylogenetic trees generated with IQ-tree, employing 15 randomly selected sequences from each flu season bin.

## Abbreviations

The following abbreviations are used in this manuscript:

ARM	Association Rule Mining
BLAST	Basic Local Alignment Search Tool
BV-BRC	Bacterial and Viral Bioinformatics Resource Center
DNA	Deoxyribonucleic Acid
EMBOSS	The European Molecular Biology Open Software Suite
HA	Hemaglutinin
IAV	Influenza A Virus
MI	Mutual Information
ML	Maximum Likelihood
NA	Neuraminidse
NCBI	National Center for Biotechnology Information
NGS	Next Generation Sequencing
RBS	Receptor Binding Site
SA	Sialic Acids
