# Supplementary material for: Evolutionary Insights from Association Rule Mining of Co-Occurring Mutations in Influenza Hemagglutinin and Neuraminidase
Source: Viruses. 2024 Sep 25;16(10):1515. doi: 10.3390/v16101515 (PMC11512220; doi:10.3390/v16101515)
Supplement: Supplementary file 1 [file viruses-16-01515-s001.zip › viruses-3159367-2final supplementary figures.pdf]

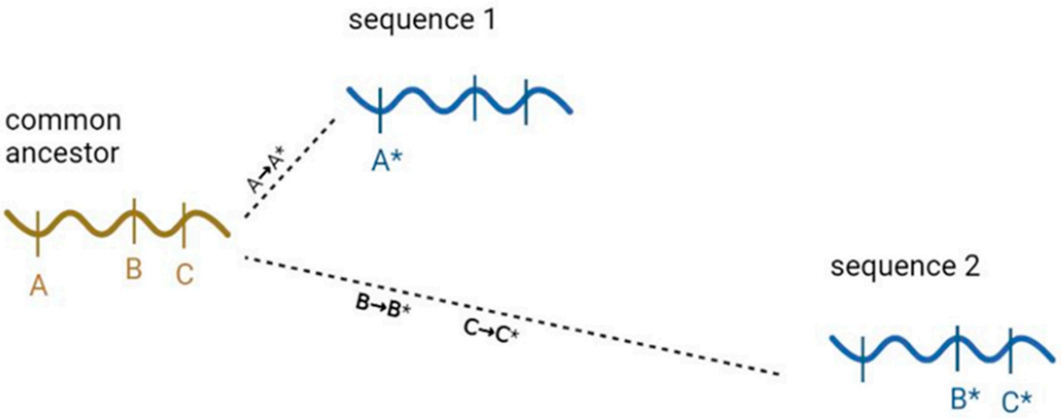

Figure S1. Illustration of potential 'reverse mutations', where sequence 1 and 2 are randomly selected from two consecutive flu seasons (bins) with sequence 2 from a more recent bin. A, B, and C are site mutations, and this example assumes that the common ancestor of the two sequences and its amino acid at sites A, B, and C are known. When the two sequence are aligned, the corresponding transaction is be represented by the following set: (A*→A, B→B*, and C→C*). In this example, mutation B→B* and C→C* are correctly detected in the transaction but since sequence 2 is considered more recent, and the mutation at A appears reversed.

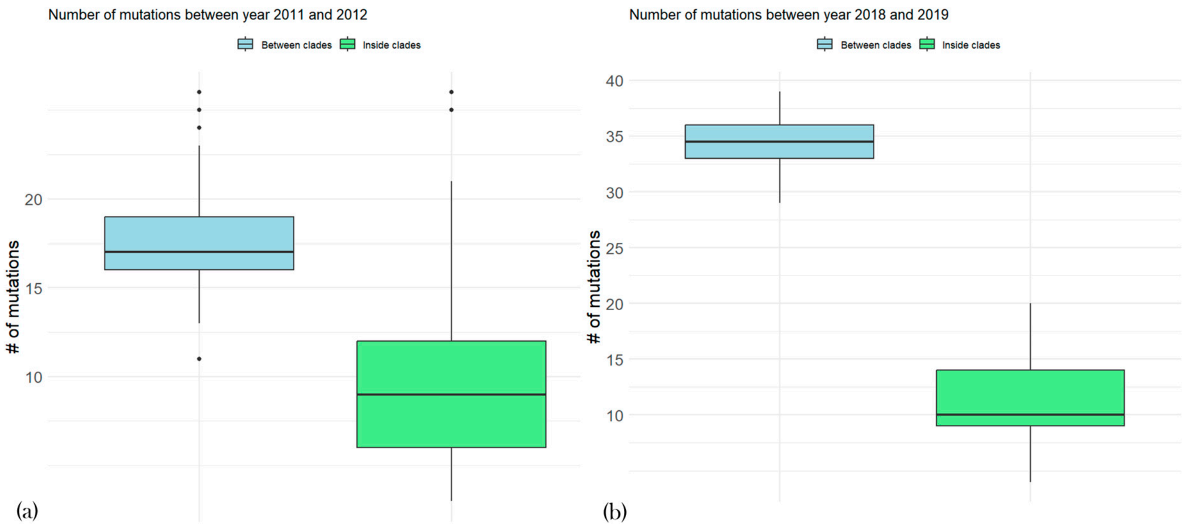

(a) (b)

Figure S2. Boxplot showing the different number of mutations when the sequences belong to the same clade compared to belonging to different clades in (**a**) 2012 and (**b**) 2019. The clades are inferred from using ML in IQ-TREE [38] by collecting the sequences that are well separated into different branches of the tree.

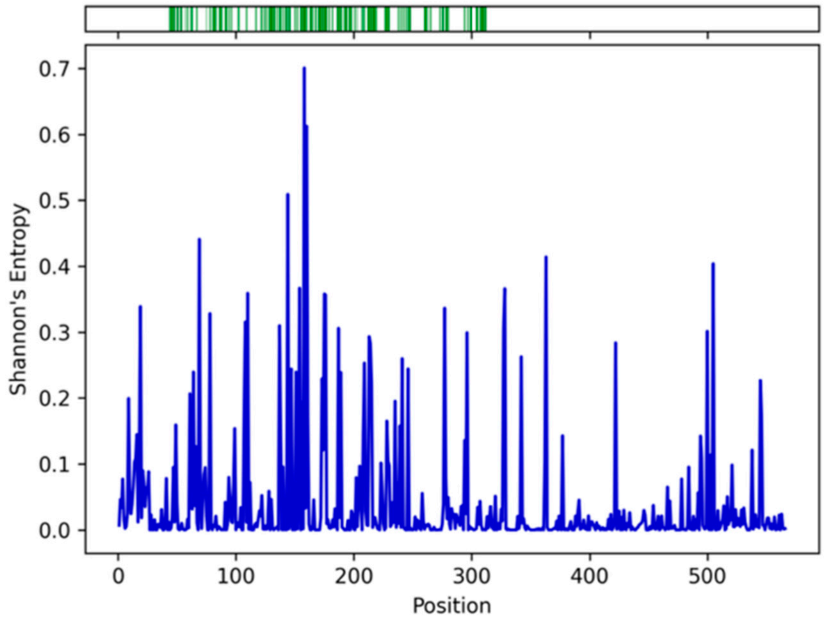

(a)

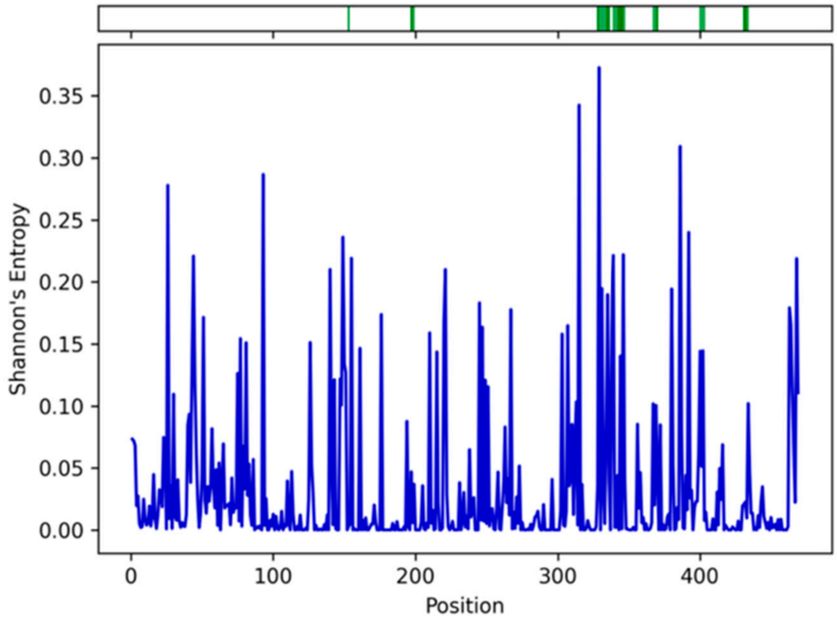

(b)

Figure S3. Mean Shannon's entropy for each amino acid residue for the H3N2 dataset (**a**) hemagglutinin, and (**b**) neuraminidase. The green bars highlight the locations of antigenic sites. Positions with a high entropy showed a larger number of amino acids in the frequency plots. The consistent alignment of peaks at similar heights and positions across these plots suggests a potential interrelation between these mutations. Figure S4 displays the frequency plots for positions 158, 160, and 144.

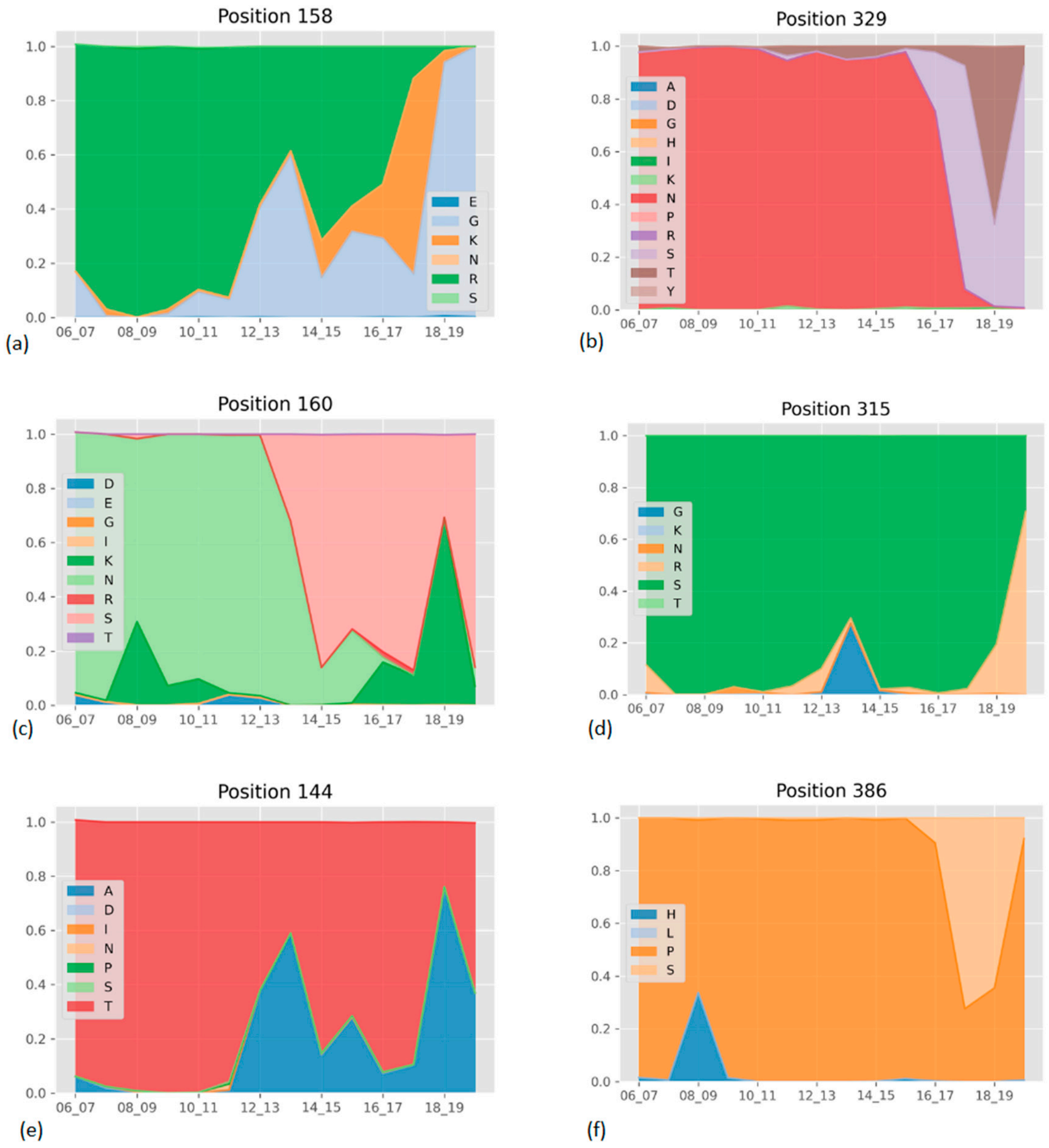

Figure S4. Frequency plots of amino acid positions depicting residue frequency for each flu season. Positions with the highest entropy for HA (**a**,**c**,**e**) and NA (**b**,**d**,**f)** are shown.

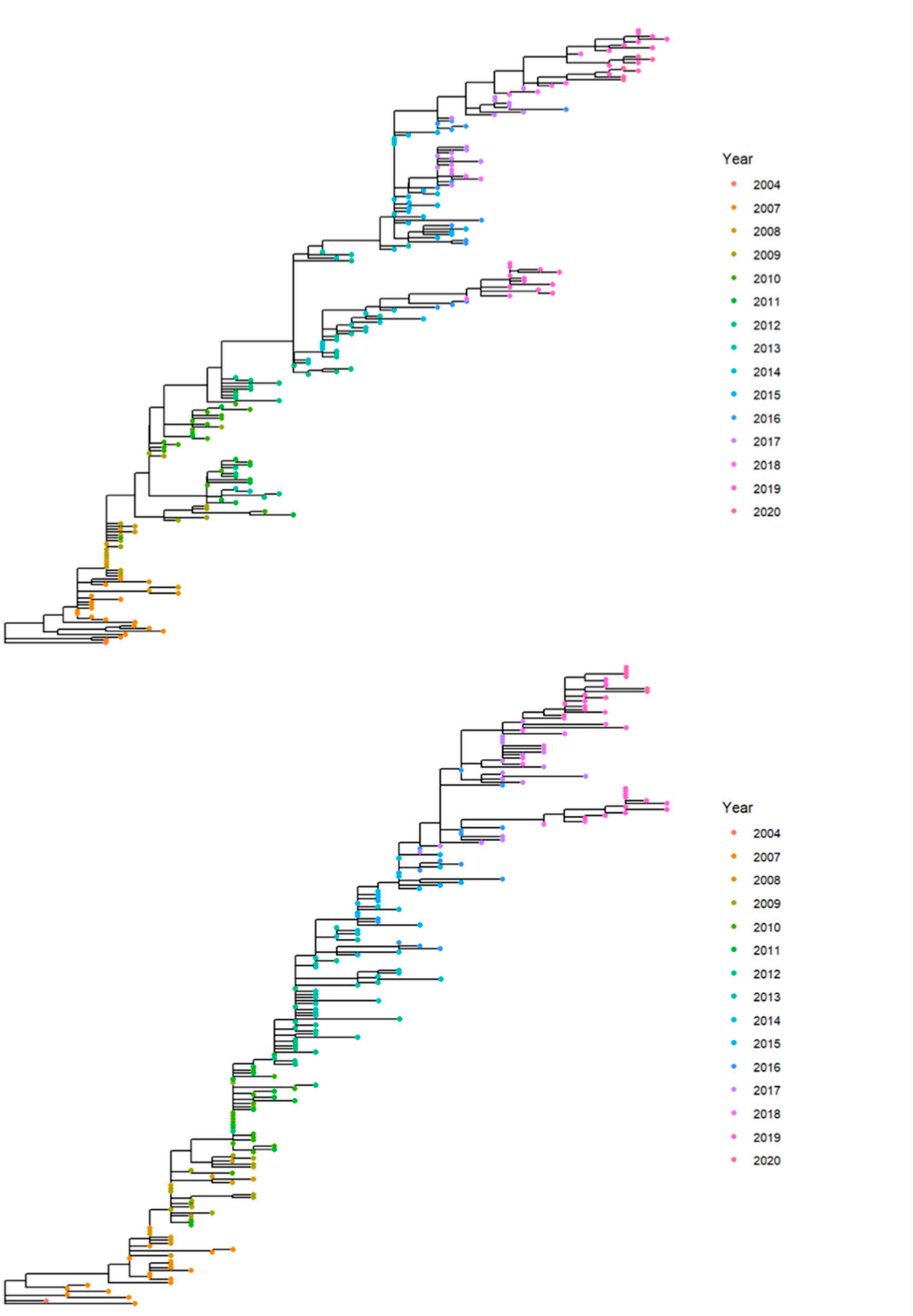

Figure S5. H3N2 maximum likelihood phylogenetic trees generated with IQ-TREE, employing 15 randomly selected sequences from each flu season bin. The top tree represents the HA gene and exhibits greater branching compared to the bottom tree representing the NA gene.
